# 

**Published:** 1858-01

**Authors:** 


					THE
AMERICAN
JOURNAL
OF
DENTAL
SCIENCE:
EDITED BY
CHAPIN A. HARRIS, M. D., D. D. S.
AND
A. SNOWDEN PIGGOT, M. D.
VOL. 8 3ST E W SERIES
PHILADELPHIA:
LINDSAY & BLAKISTON.
LONDON:
TRUBNER & CO. 12 PATERNOSTER ROW.
1858.
w
1
A
45
IA
J. W. WOODS, PRINTER,
BALTIMORE.

				

